# Indoleamine 2,3-Dioxygenase Controls Fungal Loads and Immunity in Paracoccidioidomicosis but is More Important to Susceptible than Resistant Hosts

**DOI:** 10.1371/journal.pntd.0003330

**Published:** 2014-11-20

**Authors:** Eliseu F. Araújo, Flávio V. Loures, Silvia B. Bazan, Claudia Feriotti, Adriana Pina, Alessandra S. Schanoski, Tânia A. Costa, Vera L. G. Calich

**Affiliations:** Departamento de Imunologia, Instituto de Ciências Biomédicas, Universidade de São Paulo, São Paulo, São Paulo, Brazil; University of California, San Diego, School of Medicine, United States of America

## Abstract

**Background:**

Paracoccidioidomycosis, a primary fungal infection restricted to Latin America, is acquired by inhalation of fungal particles. The immunoregulatory mechanisms that control the severe and mild forms of paracoccidioidomycosis are still unclear. Indoleamine 2,3-dioxygenase (IDO), an IFN-γ induced enzyme that catalyzes tryptophan metabolism, can control host-pathogen interaction by inhibiting pathogen growth, T cell immunity and tissue inflammation.

**Methodology/Principal Findings:**

In this study, we investigated the role of IDO in pulmonary paracoccidioidomycosis of susceptible and resistant mice. IDO was blocked by 1-methyl-dl-tryptophan (1MT), and fungal infection studied *in vitro* and *in vivo*. *Paracoccidioides brasiliensis* infection was more severe in 1MT treated than untreated macrophages of resistant and susceptible mice, concurrently with decreased production of kynurenines and IDO mRNA. Similar results were observed in the pulmonary infection. Independent of the host genetic pattern, IDO inhibition reduced fungal clearance but enhanced T cell immunity. The early IDO inhibition resulted in increased differentiation of dendritic and Th17 cells, accompanied by reduced responses of Th1 and Treg cells. Despite these equivalent biological effects, only in susceptible mice the temporary IDO blockade caused sustained fungal growth, increased tissue pathology and mortality rates. In contrast, resistant mice were able to recover the transitory IDO blockade by the late control of fungal burdens without enhanced tissue pathology.

**Conclusions/Significance:**

Our studies demonstrate for the first time that in pulmonary paracoccidioidomycosis, IDO is an important immunoregulatory enzyme that promotes fungal clearance and inhibits T cell immunity and inflammation, with prominent importance to susceptible hosts. In fact, only in the susceptible background IDO inhibition resulted in uncontrolled tissue pathology and mortality rates. Our findings open new perspectives to understand the immunopathology of paracoccidioidomycosis, and suggest that an insufficient IDO activity could be associated with the severe cases of human PCM characterized by inefficient fungal clearance and excessive inflammation.

## Author Summary

Immunoprotection to paracoccidiodomycosis, a systemic mycosis endemic in Latin America, is mediated by T cell immunity whereas immunosuppression characterizes the severe forms of the disease. Indoleamine 2,3-dioxygenase (IDO), an enzyme mainly induced by IFN-γ, catabolizes tryptophan along the kynurenines pathway. Tryptophan deficiency has been associated with reduced pathogen growth, while elevated levels of kynurenines with suppressed immune responses. In this study, the role of IDO in pulmonary paracoccidioidomycosis was investigated using resistant and susceptible mice. In both mouse strains, IDO blockade by 1-methyl tryptophan resulted in inefficient fungal clearance accompanied by enhanced T cell immunity. Despite these equivalent biological effects, only in susceptible mice IDO inhibition caused progressive fungal growth and tissue pathology resulting in increased mortality. Our findings demonstrate for the first time that IDO exert a yet unexplored immunoregulatory role in pulmonary paracoccidioidomycosis that can be particularly important in the severe cases of the disease.

## Introduction

Indoleamine-2,3-dioxygenase (IDO) is the major and rate limiting cytosolic enzyme of tryptophan catabolism along the kynurenines pathway [1] and is expressed primarily in macrophages, epithelial and dendritic cells (DCs). The enzyme is induced by proinflammatory cytokines (e.g., IFN-γ), Toll-like receptor ligands (e.g., lipopolysaccharide), and interactions between immune cells (e.g., the engagement of costimulatory molecules on antigen-presenting cells by cytotoxic T-lymphocyte antigen-4) [2], [3], [4], [5]. Albeit IDO is critical to host defense against pathogens, it is now clear that this enzyme performs multiple roles in the immune system mainly by interfering with the T cell proliferative capacity [5], [6], [7]. Some immunoregulatory pathways that are induced by the decreased levels of tryptophan could be linked with inhibition of mammalian target of rapamycyn (mToR) signaling and activation of the amino acid sensitive general control nonderepressible 2 (GCN2) stress kinase pathway, that can cause cell cycle arrest, anergy and apoptosis of responding T cells [5], [8], [9]. Increased tryptophan catabolism by IDO suppresses T cell responses in a variety of diseases or states, including autoimmune disorders [10], allograft rejection [11], viral infections [12], [13], cancer [14], and pregnancy [15].

IDO activity can be host protective by suppressing pathogen replication [16], [17], [18], but can also suppress host immunity resulting in augmented pathogen burdens. Infected hosts can use the reduced inflammatory or allergic responses induced by IDO to improve immunoprotection. In experimental *Candida albicans* infection, the upregulated IDO expression and increased kynurenines production were shown to control fungal burdens and Th1/Treg cells development, inhibiting deleterious Th17 immunity. Indeed, IDO blockade with 1-methyl-dl-tryptophan (1MT) favors fungal persistence and IL-23/Th17 axis of inflammation, linking inadequate immune response to chronic inflammation [19], [20], [21]. IDO was also shown to regulate the host response to *Aspergillus sp* by activating distinct populations of Treg cells, which suppress proinflammatory neutrophils in the early phase and prevent allergic responses in the late phase of infection [22]. Thus, IDO appears to have evolved to control pathogen persistence while preventing extensive tissue pathology in an inflammatory host environment [23], [24].

Paracoccidioidomycosis (PCM), a systemic granulomatous disease caused by the dimorphic fungus *Paracoccidioides brasiliensis*, constitutes the most prevalent deep mycosis in Latin America [25]. The inhalation of conidia usually leads to an asymptomatic infection but a few infected individuals evolve to overt disease. The infection and the benign forms of the disease are associated with prevalent Th1 immunity whereas the severe forms are allied with suppressed DTH responses and prevalent Th2/Th3 immunity [26], [27]. Our laboratory has established a murine model of pulmonary PCM. In this model, A/J and B10.A mice are resistant and susceptible to *P. brasiliensis* infection, respectively. The A/J mice develop chronic, benign pulmonary-restricted PCM, coupled with well-organized lesions containing a low number of yeasts and positive delayed-type hypersensitivity (DTH) reactions resembling those in the benign form of PCM. In contrast, B10.A mice develop a progressive disseminated disease associated to increased fungal loads, DTH anergy, and non-organized lesions mimicking those in the severe forms of PCM [28], [29], [30]. Both CD4^+^ and CD8^+^T cells were shown to be immunoprotective against *P. brasiliensis* infection. Resistant mice develop mixed Th1/Th2 responses concomitant with protective CD8^+^ T cells that synthesize large amounts of IFN-γ [31], [32], [33]. The relative protection of susceptible mice is mediated by CD8^+^ T cells which, however, are not able to compensate the CD4^+^ T cell anergy induced by excessive proinflammatory innate response [31], [33], [34], [35], [36]. The present study was designed to characterize the role of IDO and its metabolites in the disease developed by susceptible and resistant mice to *P. brasiliensis* infection. Our findings provide evidence that IDO is an important immunoregulatory enzyme for both mouse strains. Employing 1MT, an easily available drug that inhibits IDO-1 and IDO-2 [16], we could verify that *P. brasiliensis* infection induces increased IDO activity that reduces fungal loads and T cell immunity concurrently with increased expansion of Treg cells. The suppressive effect of IDO was mainly directed towards several subsets of IL-17 producing T cells that occurred with simultaneous up-regulation of IFN-γ producing T cells. Although in both mouse strains IDO was shown to exert similar regulatory effects, significantly increased tissue pathology and mortality rates were only observed in 1MT treated susceptible mice suggesting that IDO plays a more important regulatory role in the severe forms of the disease.

## Materials and Methods

### Ethics statement

Animal experiments were performed in strict accordance with the Brazilian Federal Law 11,794 establishing procedures for the scientific use of animals, and the State Law establishing the Animal Protection Code of State of São Paulo. All efforts were made to minimize suffering, and all animal procedures were approved by the Ethics Committee on Animal Experiments of the Institute of Biomedical Sciences of University of São Paulo (Proc.76/04/CEEA).

### Mouse strains

Susceptible (B10.A) and resistant (A/J) mouse strains to *P. brasiliensis* infection were obtained from our Isogenic Unit (Immunology Department of Institute of Biomedical Sciences of University of São Paulo, Brazil) and used at 8 to 11 weeks of age. Specific pathogen free mice were fed with sterilized laboratory chow and water *ad libitum*.

### Fungus

*P. brasiliensis* 18 (Pb 18) was used throughout this investigation. Pb18 yeast cells were maintained by weekly sub cultivation in semisolid Fava Netto culture medium [37] at 37°C and used on the seventh day of culture. Phosphate buffered saline (PBS)-washed yeast cells were adjusted 4×10^4^ cells/ml based on hemocytometer counts. Viability was determined with Janus Green B vital dye (Merck) and was always higher than 85%.

### Fungicidal assay

Thioglycollate-induced peritoneal macrophages were isolated by adherence (2 h at 37°C in 5% CO_2_) to plastic-bottom tissue-culture plates (1×10^6^ cells/well in 24 well plates). Macrophages were washed to remove nonadherent cells and cultivated overnight with fresh complete medium (DMEM, Dulbecco's Modified Eagle's Medium, Sigma, containing 10% fetal calf serum, 100 U/ml penicillin and 100 µg/ml streptomycin) in the presence or absence of recombinant IFN-γ (20 ng/ml in culture medium, BD-Pharmingen) and/or 1MT (1-methyl-d,l-tryptophan, Sigma-Aldrich). 1MT was used in the concentration of 1 mM that was previously shown to efficiently inhibit the IDO activity of macrophages [6]. Macrophage cultures were infected or not with *P.brasiliensis* yeasts in a macrophage∶yeast ratio of 25∶1 and cocultivated for 4 h. This ratio was previously determined and was shown to be non-deleterious to macrophage cultures and adequate for killing assays [38]. The monolayers were then washed to remove nonadherent cells and incubated for an additional 48 h period in the presence or absence of IFN-γ (20 ng/ml in culture medium, BD Biosciences) and/or 1MT.

### CFU assay

Coculture homogenates (100 µl) were plated on brain heart infusion agar (Difco laboratories), containing 4% (v/v) horse serum (Instituto Butantan, São Paulo, Brazil) and 5% *P. brasiliensis* 192 culture filtrate, the latter constituting the source of growth-promoting factor [39]. When necessary, dilutions were made in sterile PBS. The plates were incubated at 37°C, and colonies were counted daily until no increase in counts was observed. The numbers (log10) of viable *P. brasiliensis* colonies are expressed as the means ±SE.

### 1MT treatment and *in vivo* fungal infection

To ensure the maintenance of its virulence, the isolate was used after three serial animal passages. *P. brasiliensis* yeast were washed in PBS, counted and adjusted to 20×10^6^ fungal cells/ml. Mice were anesthetized and submitted to intra-tracheal (i.t.) *P. brasiliensis* infection as previously described [40]. Briefly, after intra-peritoneal (i.p.) anesthesia the animals were infected with 1×10^6^ Pb18 yeast cells, contained in 50 µl of PBS, by surgical i.t. inoculation, which allowed dispensing of the fungal cells directly into the lungs. The skins of the animals were then sutured, and the mice were allowed to recover under a heat lamp. At the same day, groups of B10.A and A/J mice were treated with i.p. injections of 5 mg/ml of 1MT or 1 mg/ml of rice starch (Sigma-Aldrich) as control. Mice were treated during 2 weeks and sacrificed 2 and 8 weeks after infection. Alternatively, slow-release polymer pellets (Innovative Research of America, 5 mg/day) containing 1MT or vehicle alone were inserted under the dorsal skin of B10.A and A/J mice [15], and mice were sacrificed 4 and 8 weeks after *P. brasiliensis* infection. Each pellet contained 150 mg of 1MT designed to continuously release 5 mg per day during 30 days. The pellet was composed by a biodegradable matrix that effectively and continuously released the active product in the animal. The concentration of 5 mg of 1MT/day by the i.p. route was used because previous reports have demonstrated that this schedule was sufficient to efficiently inhibit IDO activity [41]. Two to three experiments were performed separately.

### Assays for tissue CFU, mortality rates, and histological analysis

The numbers of viable microorganisms in infected organs (lungs and liver) from experimental and control mice were determined by counting the number of CFU. Animals from each group were sacrificed, and the enumeration of viable organisms was done as previously described [39]. The numbers (log10) of viable *P. brasiliensis* per gram of tissue are expressed as the means ± standard errors. Mortality studies were performed with groups of 9 or 10 mice inoculated i.t. with 1×10^6^ yeast cells or PBS. Deaths were registered daily, and experiments were repeated twice. For histological examinations, the left lung of the infected mouse was removed, fixed in 10% formalin, and embedded in paraffin. Five-micrometer sections were stained by hematoxylin-eosin (H&E) for analysis of the lesions and silver stained (Grocott stain) for fungal evaluation. Pathological changes were analyzed based on the size, morphology, cell composition of granulomatous lesions, the presence of fungi, and the intensity of the inflammatory infiltrates. Morphometric analysis was performed using a Nikon DXM 1200c digital camera (magnification of ×100) and Nikon NIS Elements AR 2.30 software. The area of lesions (in µm^2^) was measured using 10 microscopic fields per slide for 6 mice per group. Results were expressed as the means ± standard errors for the total area of lesions for each animal.

### NO and cytokines measurement

Supernatants from cell cultures and lung homogenates were separated and stored at −70°C. The levels of IL-6, IL-12, IL-10, IL-4, IFN-γ, IL-23, IL-17, tumor necrosis factor alpha (TNF-α), and tissue growth factor-beta (TGF-β) were measured by a capture enzyme-linked immunosorbent assay (ELISA) with antibody pairs purchased from eBioscience. The ELISA procedure was performed according to the manufacture's protocol, and absorbance was measured with a Versa Max Microplate Reader (Molecular Devices). The concentrations of cytokines were determined based on a standard curve of serial twofold dilutions of murine recombinant cytokines. Nitric oxide production was quantified by the accumulation of nitrite in the supernatants from *in vitro* protocols by a standard Griess reaction [42]. All determinations were performed in duplicate, and results were expressed as micro molar concentration of NO.

### Determination of IDO enzymatic activity

To monitor IDO enzymatic activity, kynurenines were measured using a modified spectrophotometric assay [43] The amount of 50 µl of 30% trichloroacetic acid was added to 100 ml of lung homogenates or macrophages cultures supernatants, vortexed, and centrifuged at 800 *g* for 5 min. A volume of 75 µl of the supernatant was then added to an equal volume of Ehrlich reagent (100 mg P-dimethylbenzaldehyde, 5 ml glacial acetic acid) in a 96 well microtiter plate. Optical density was measured at 492 nm, using a Multiskan MS (Labsystems) microplate reader. A standard curve of defined kynurenine concentrations (0–100 mM) was used to determine unknown kynurenine concentrations.

### Quantitative analysis of IDO mRNA expression

RNA was extracted from macrophages cultures infected or not with *P.brasiliensis*, treated or not with 1MT, and from lungs of uninfected and infected mice, treated or not with 1MT. The RNA concentrations were determined by spectrophotometer readings at an absorbance of 260 nm. First-strand cDNAs were synthesized from 2 µg RNA using the High Capacity RNA-to-cDNA kit (Applied Biosystems) according to the manufacturer's instructions. Real-time polymerase chain reaction (RT-PCR) specific for IDO (primer Mm00492586_m1, Applied Biosystems, Life Technologies) was performed using the TaqMan real-time PCR assay (Applied Biosystems, Life Technologies) according to the manufacturer's instructions. Analysis was performed with the ABI PRISM 7000 sequence detection system (Applied Biosystems). GAPDH was used as an internal control. All values were normalized to GAPDH, and the relative gene expression calculated.

### Flow cytometry

For cell surface staining, lung leukocytes were washed and resuspended at a concentration of 1×10^6^ cells/ml in staining buffer (PBS 1×, 2% FBS, 0,5% NaN_3_). Fc receptors were blocked by the addition of unlabeled anti-CD16/32 (Fc block; BD Biosciences). The leukocytes were then stained for 30 min at 4°C with the optimal dilution of each antibody labeled with the adequate fluorochrome (BD Biosciences). Phycoerythrin (PE)-labeled anti-IA^K^ (clone11-5.2), anti-CD44 (clone IM7), anti-B220 (clone RA3-6B2), and fluorescein isothiocyanate (FITC) anti-CD69 (clone H1.2F3), and Alexa Fluor 488 anti-GITR (clone YGITR 765), and PE-Cy7 anti-CD11c (clone N418), and peridinin chlorophyll protein (PerCP) complex-Cy5.5 anti-CD4 (clone RM4-5), anti-CD40 (clone 3/23), Pacific Blue anti-CD11b (clone M1/70), anti-CD8 (clone 53-6.7), and allophycocyanin (APC) anti-CD86 (clone GL1), anti-CTLA-4 (clone UC10-4B9), and APC-Cy7 anti-CD62L (clone MEL-14) monoclonal antibodies (MAbs; BD Biosciences) were used. Cells were washed twice with staining buffer, resuspended in 100 µl, and an equal volume of 2% formaldehyde was added to fix the cells. The stained cells were analyzed on a FACSCanto flow cytometer (BD Biosciences) using the FACSDiva software (BD Biosciences). LIL lymphocytes were identified on forward-scatter (FSC) and side-scatter (SSC) analysis. Gated cells were measured for CD4^+^ and CD8^+^ expression followed by CD44 expression, and cells expressing high and low levels of this molecule were gated. Gated CD44^high^ cells were then measured for expression of low levels of CD62L identifying the effector/memory CD4^+^CD44^high^CD62L^low^ and CD8^+^CD44^high^CD62L^low^ subpopulation. Gated CD44low cells were then measured for the expression of high levels of CD62L identifying the naive CD4^+^CD44^low^CD62L^high^ and CD8^+^CD44^low^CD62L^high^ subpopulation. Gated CD44^low^ cells were then measured for the expression of high levels of CD62L identifying the naive CD4^+^CD44^low^CD62L^high^ subpopulation. Fifty thousand cells were acquired and the data expressed as the absolute number of positive cells, which was calculated from the percentage obtained by flow cytometry and the number of cells determined in Neubauer chambers.

### Assessment of leukocyte populations

For differential counts, samples of lung cell suspensions were cytospin (Shandon Cytospin) onto glass slides and stained by the Diff-Quik blood stain (Baxter Scientific). A total of 200 to 400 cells were counted from each sample. The absolute number of a leukocyte subset was calculated by multiplying the percentage of each subset in an individual sample by the total number of lung leukocytes in that mouse.

#### Characterization of intracellular cytokines and FoxP3

Flow cytometry was used to measure the expression intracellular FoxP3, IFN-γ, IL-17 and IL-4. For surface molecules, PE-Cy7 anti-CD4, Pacific Blue anti-CD8α, PE anti-γδ (clone 17A2), PE anti-NKT (clone 37.51) and FITC anti-CD25 (clone 7D4), from BD Biosciences were used. For intracellular cytokine staining, lung cells were stimulated for 6 h in complete RPMI medium in the presence of 50 ng/ml phorbol 12-myristate 13-acetate, 500 ng/ml ionomycin (both from Sigma-Aldrich) and brefeldin A (eBioscience). LIL lymphocytes were identified on forward-scatter (FSC) and side-scatter (SSC) analysis. Gated cells were measured for CD4^+^, CD8^+^, NKT^+^ and γδ^+^ expression. Gated cells were then measured for IL-17, IL-4 and IFN-γ positive CD4^+^, CD8^+^, NKT^+^ and γδ^+^ T cells. Treg cells were characterized by intracellular staining for FoxP3 (PE, clone NRRF-30), using the Treg staining kit of eBioscience. LIL lymphocytes were identified on FSC and SSC analysis. Gated cells were measured for CD4 expression and then for CD25 expression. Gated CD4^+^CD25^+^ cells were measured for Foxp3 expression, identifying Tregs. To measure the expression of intracellular IFN-γ, IL-17 and IL-4, after staining of surface molecules, cells were fixed, permeabilized and stained by APC anti-IFN-γ (clone XMG1.2), FITC anti-IL-4 (clone BVD6-2462) and PercP anti-IL-17 (clone eBio1787) antibodies (BD Bioscience or eBioscience). The cell surface expression of leukocyte markers as well as intracellular cytokine expression was analyzed using the FlowJo software (Tree Star).

### Statistical analysis

Data are expressed as the mean ± SEM. Differences between groups were analyzed by Student's *t* test or analysis of variance (ANOVA) followed by the Bonferroni test. Differences between survival times were determined with the LogRank test. Data were analyzed using GraphPad Prism 6.0 software for Windows (GraphPad). A *p* value of ≤0.05 was considered statistically significant.

## Results

### 1MT treatment increases the fungal loads but reduces kynurenines and IDO mRNA production by *P. brasiliensis* infected macrophages

**Figure 1:**
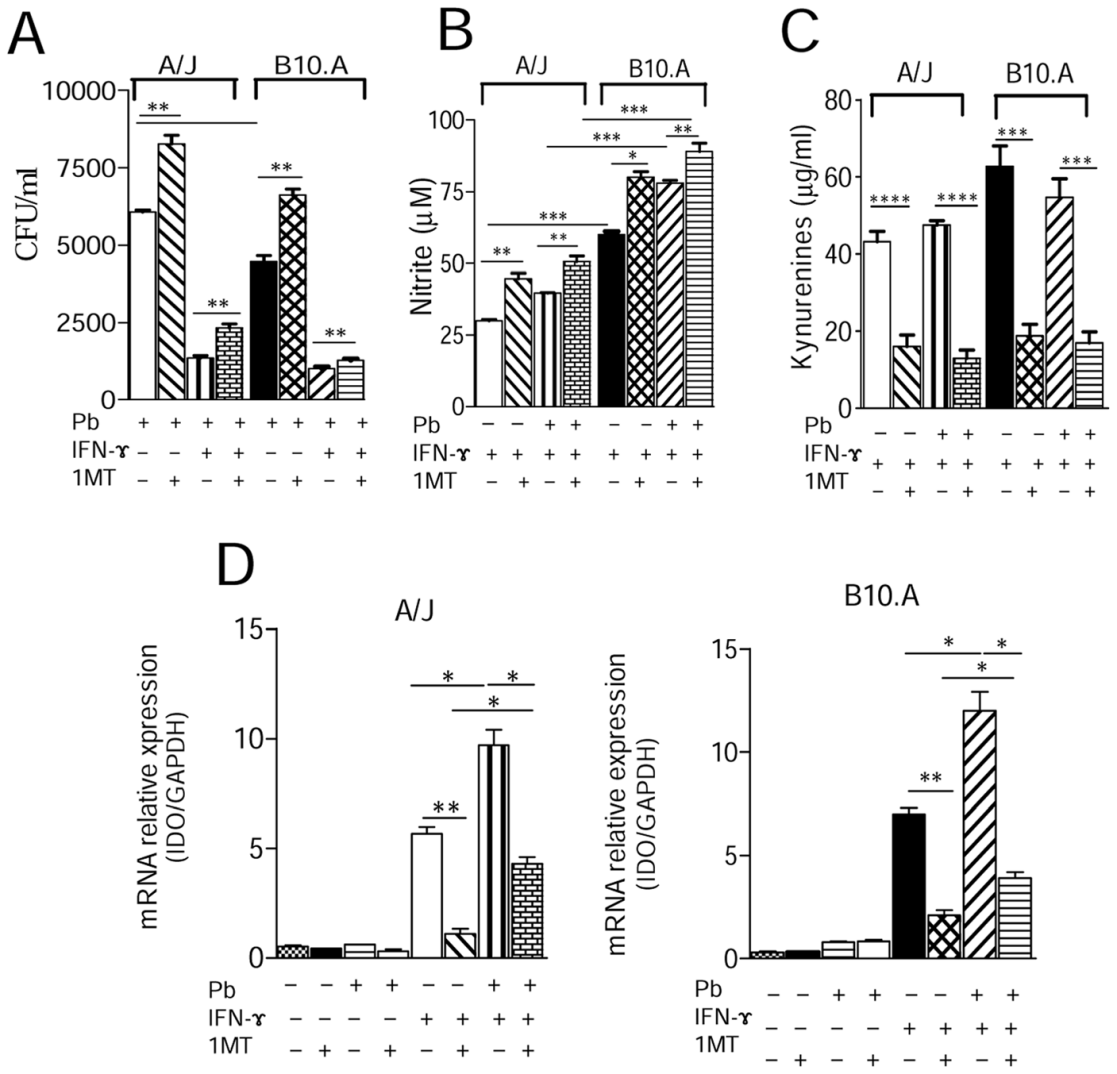
IDO Inhibition increases the fungal loads of macrophages and inhibits kynurenines and IDO mRNA production. (**A**) IFN-γ-primed and unprimed peritoneal macrophages from A/J and B10.A mice strains were treated with 1MT (1 mM) or left untreated and subsequently infected with *P. brasiliensis* yeasts in a macrophage∶yeast ratio of 25∶1 for 2 h. Infected and uninfected macrophages were then cultivated for 48 h at 37°C in 5% CO2. The monolayers were lysed with distilled water, and 100 µl of cell homogenates were assayed for the presence of viable yeasts by a CFU assay. (**B, C**) Supernatants from macrophage cultures were used to determine the levels of nitrite and kynurenines. (**D**) In the same experimental conditions, peritoneal macrophages of A/J and B10.A mice were mixed with TRizol reagent for RNA extraction and then submitted to qRT-PCR assays for mRNA IDO evaluation (**D**). Data are the means ± SEM of quintuplicate samples from one experiment representative of 3 independent determinations (*p<0.05, **p<0.01, ***p<0.001 and ****p<0.0001).

Initially we sought to investigate the effects of IDO inhibition in the interaction between macrophages from susceptible and resistant mice with *P. brasiliensis*. To do so, macrophages were treated with IFN-γ and/or 1MT, or left untreated, and then cultured or not with *P. brasiliensis*. The 1MT treatment led to increased recovery of viable yeast cells from untreated and IFN-γ-primed macrophages in both mouse strains (Figure 1A). Unexpectedly, the increased fungal loads were concomitant with increased production of NO, the most known *P. brasiliensis* fungicidal mediator [44]. As observed in our previous studies [35], macrophages from B10.A mice (1MT treated and untreated) produced higher levels of NO than those of A/J mice (Figure 1B). Inhibition of IDO by 1MT led to diminished levels of kynurenines production by macrophages from both mouse strains (Figure 1C). We further analyzed by quantitative PCR the expression of IDO mRNA by uninfected and infected macrophages. Cells from both mouse strains showed diminished IDO mRNA levels after treatment with 1MT (1D), which is in agreement with the kynurenine concentrations observed in Figure 1C.

### 1MT treatment reduces IL-12 but increases IL-6 and TGF-β production by *P. brasiliensis* infected macrophages

**Figure 2:**
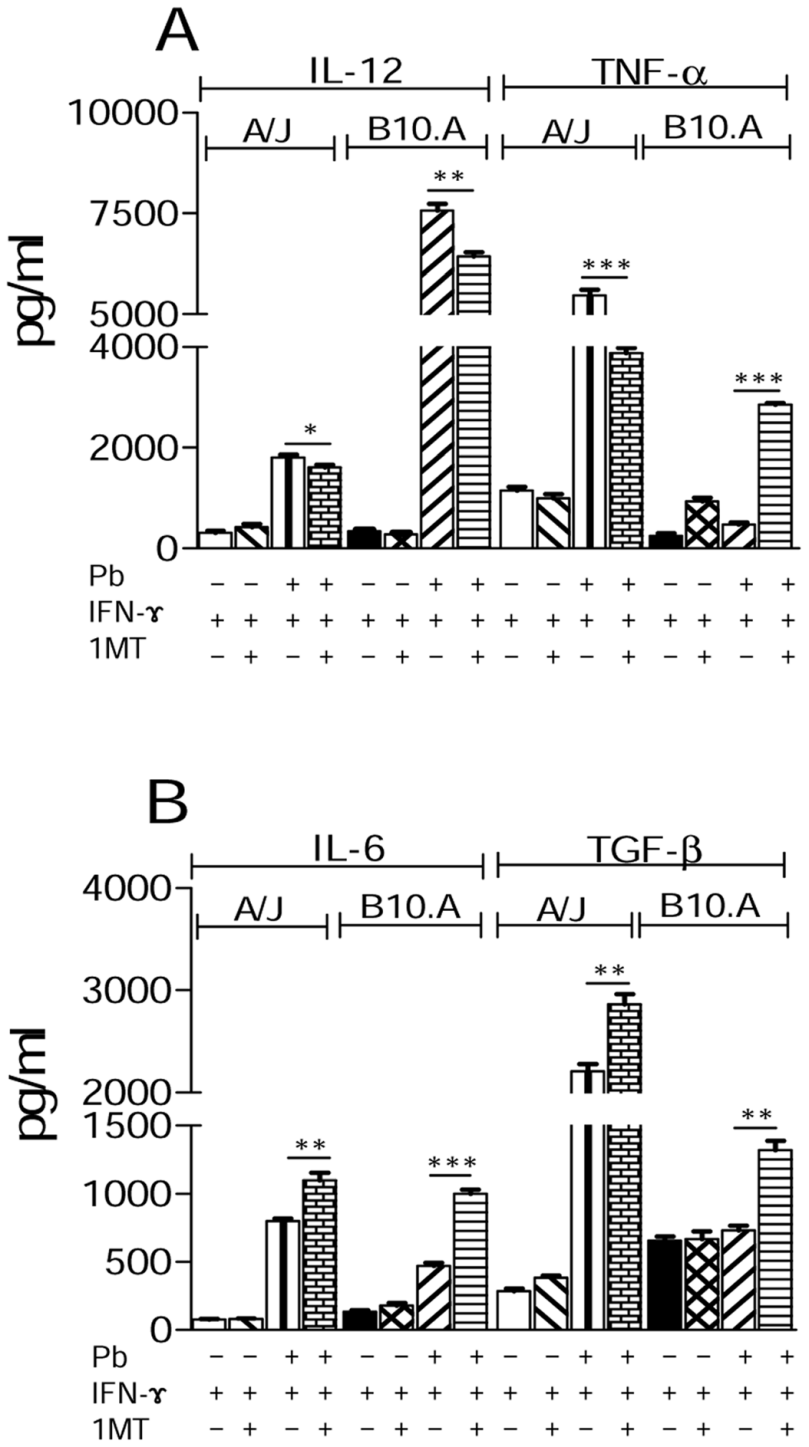
1MT treatment reduces IL-12 and increases IL-6 and TGF-β production by A/J and B10.A macrophages. (**A, B**) IFN-γ-primed and unprimed macrophages were treated with 1MT or left untreated and then cultivated for 48 h. Some cultures were infected with viable *P. brasiliensis* yeasts (1∶25, fungus∶macrophages ratio) for 48 h. Supernatants were removed and used for cytokine measurements by means of ELISA. Data are means ± SEM of triplicate samples from three independent determinations (*p<0.05, **p<0.01 and ***p<0.001).

When cytokines in macrophage culture supernatants were measured, reduced IL-12 levels were observed in 1MT-treated macrophages of both mouse strains. Interestingly, TNF-α secretion by macrophages from A/J mice decreased significantly after 1MT treatment, whereas B10.A macrophages produced higher amounts of this cytokine than their untreated counterparts (Figure 2A). We also observed an expressive increase of IL-6 and TGF-β in 1MT treated A/J and B10 macrophages (Figure 2B).

### IDO inhibition increases the fungal loads of susceptible and resistant mice and down regulates kynurenines and IDO mRNA production

**Figure 3:**
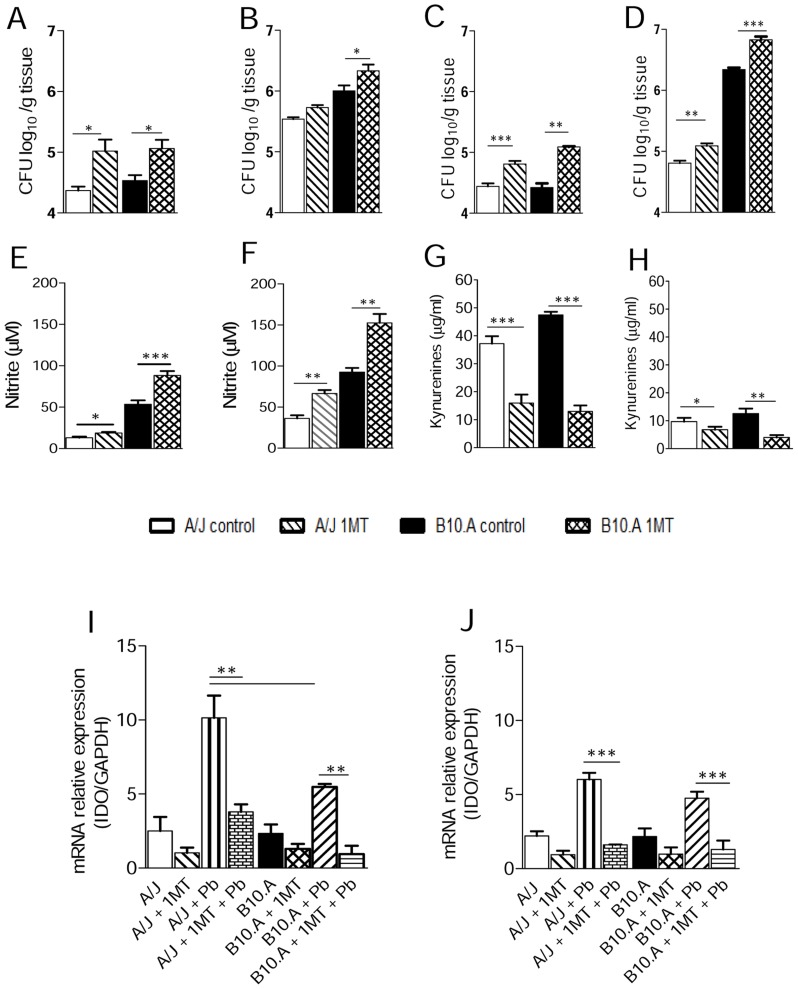
In pulmonary paracoccidioidomycosis, 1MT treatment increases fungal loads and pulmonary NO but reduces kynurenines levels and IDO mRNA production. (**A, B**) Determination of fungal loads by CFU assays in the lungs of A/J and B10.A mice (n = 5–6), treated or not with 1MT, at weeks 2 and 8 after infection with 1×10^6^ yeasts. (**C, D**) Groups (n = 6–8) of B10.A and A/J mice received subcutaneous implants of 1MT releasing or control pellets (5 mg/ml/day) and disease severity assessed by CFU assays at weeks 4 and 8 after infection with 1×10^6^ yeast cells of *P. brasiliensis*. The bars depict means ± SEM of log10 CFU. The results are representative of 2 experiments with equivalent results (**p<0.01, and ***p<0.001). Levels of (**E, F**) nitric oxide and (**G, H**) kynurenines were measured in lung homogenates. NO production was measured by Griess reagent, and kynurenines were evaluated using Ehrlich's reagent. (**I, J**) IDO mRNA was measured using TaqMan real-time PCR assay. Data are means ± SEM of three independent experiments at weeks 2 and 8 after infection with similar results (*p<0.05, **p<0.01 and ***p<0.001).

In order to explore the effect of IDO inhibition *in vivo*, A/J and B10.A mice were treated with 1MT and subsequently subjected to i.t infection with *P. brasiliensis* yeasts cells. At week 2, in both mouse strains the pulmonary infection was significantly higher in 1MT treated mice comparing to their control groups, as seen in the CFU assays (Figure 3A). As previously described [40], at week 8 the infection was more severe in susceptible than in resistant mice. Of note, increased fungal loads remained higher in 1MT treated than untreated B10.A mice, but the same behavior was not observed in the resistant strain where the fungal loads returned to control levels (Figure 3B). However, when mice were submitted to IDO inhibition using subcutaneous pellets that released 1MT during 30 days, increased CFU counts were observed in both B10.A and A/J mice at weeks 4 and 8 of infection (Figure 3C, 3D). Consistent with *in vitro* results, at weeks 2 and 8 weeks after infection the levels of NO in the lungs of 1MT treated mice from both strains was significantly higher than those detected in untreated groups (Figure 3E, 3F). Moreover, inhibition of IDO by 1MT led to diminished levels of kynurenines at week 2 and 8 postinfection (Figure 3G, 3H). We have also analyzed by quantitative PCR the expression of IDO mRNA in B10.A and A/J mouse lungs. In agreement with the reduced levels of kynurenines observed (Figure 3G, 3H), a diminished expression of IDO mRNA was found in the1MT treated groups of both mouse strains (Figure 3I, 3J).

### IDO inhibition increases pulmonary inflammation and mortality of susceptible but not resistant mice

**Figure 4:**
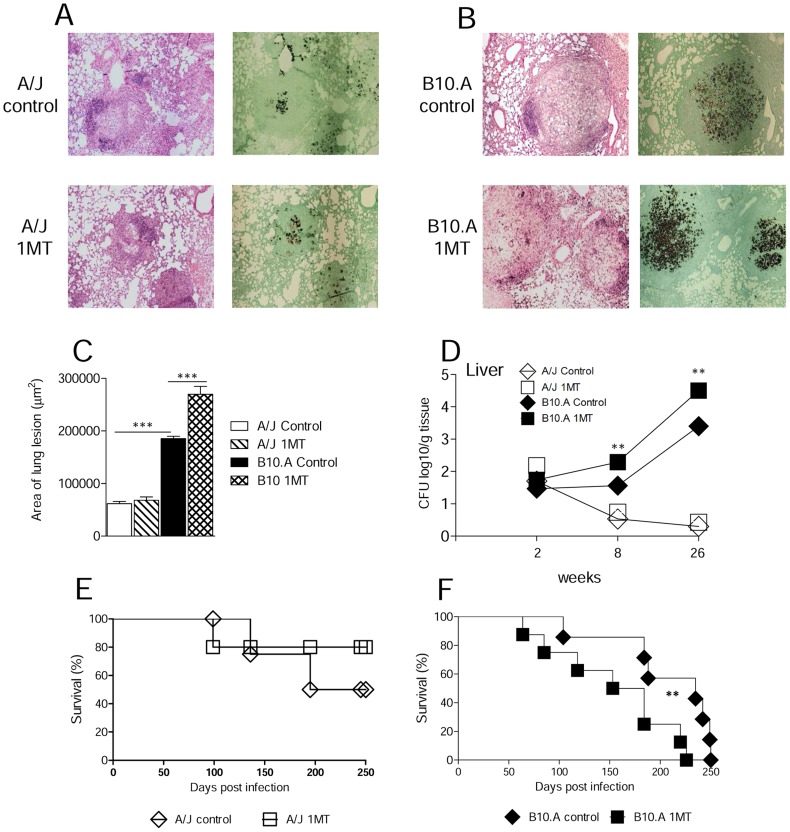
1MT treatment increases fungal loads, tissue pathology and mortality rates of susceptible but not resistant mice to *P. brasiliensis* infection. Photomicrographs of lungs from 1MT treated and untreated A/J (**A**) and B10.A (**B**) mice at week 8 after infection with 1×10^6^ *P. brasiliensis* yeast cells obtained from groups of 5–6 mice. For panels: **A** and **B**, HE-stained, magnification ×10, Grocott-stained, magnification ×10. (**C**) Morphometric analysis of lung lesions of 1MT treated and untreated B10.A and A/J mice at week 8 post-infection (n = 5–6). (**D**) At weeks 2, 8 and 26 after infection, the presence of viable yeasts was determined in the liver of 1MT treated and untreated B10.A and A/J mice (n = 5–6). (**E, F**) Survival times of 1MT treated or untreated A/J and B10.A mice (9–10 mice per group) infected with 1×10^6^ *P. brasiliensis* yeasts were determined for a period of 250 days. The results are representative of two independent experiments (**p<0.01; ***p<0.001).

The histopathology of lungs at week 8 of infection showed that control and 1MT treated A/J mice displayed reduced numbers of small, well-defined granulomas. The numbers of fungi were low, and sometimes absent from the lesions (Figure 4A). Compared with A/J mice, control B10.A mice exhibited higher numbers of large granulomas containing large amounts of fungi that affected larger areas of lungs. Furthermore, in comparison with untreated controls 1MT treated B10.A mice showed more severe lesions, composed of isolated or confluent granulomas containing a large number of budding yeast cells, involving extensive areas of lung parenchyma (Figure 4B). The total area of lesions was quantified in histological sections and shown in Figure 4C. At week 8, the lesion areas of 1MT treated B10.A mice were significantly larger than those of B10.A control mice. Whilst the lesion areas from A/J mice were smaller and showed no difference upon treatment, the histopathological studies demonstrated that administration of 1MT to susceptible mice resulted in increased fungal burdens that evolved with enhanced tissue inflammation. Further, as dissemination and control of fungal growth in the liver appear to be an important marker of the regressive and progressive infections of A/J and B10.A mice [40], [45], the fungal burden in the liver was determined. The results of CFU assays showed a significant increase in the number of viable yeasts in the1MT treated B10.A mice when compared with control mice, at weeks 8 and 26 after infection. Again, no differences in the fungal loads of A/J mice, treated or not with 1MT, were observed (Figure 4D). To further assess the influence of 1MT treatment on the disease outcome, mortality of treated and untreated *P.brasiliensis* infected A/J and B10.A mice was registered daily after infection with 1×10^6^ yeast cells. 1MT treatment led to higher mortality of susceptible mice compared with control B10.A mice whereas no significant differences were observed in A/J mice, where 50% of control and 80% of 1MT treated resistant mice were still alive after 250 days of infection (Figure 4E, 4F).

### IDO blockade promotes increased influx of dendritic cells to the lungs of susceptible and resistant mice

**Figure 5:**
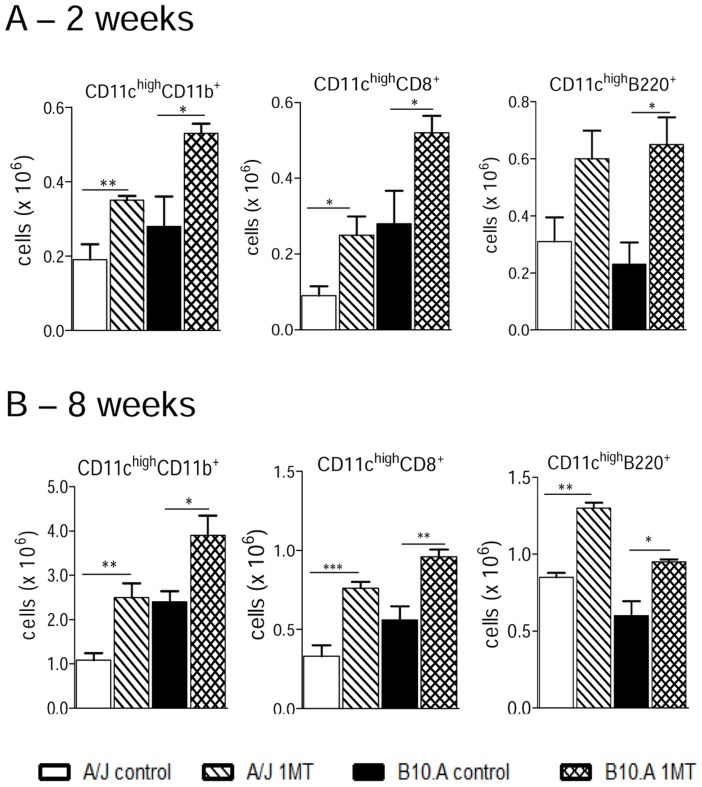
IDO inhibition increases the influx of myeloid (CD11c^high^CD11b^+^), lymphoid (CD11c^high^CD8^+^) and plasmacytoid (CD11c^high^B220^+^) dendritic cells to the lungs of A/J and B10.A mice. 1MT treated or untreated B10.A and A/J mice were infected i.t. with 1×10^6^ yeast cells of *P. brasiliensis*, and at 2 (**A**) and 8 (**B**) weeks after infection, lung inflammatory cells were obtained and lung DC subsets characterized using specific antibodies. Cell phenotypes were analyzed by flow cytometry as described in Materials and Methods. The data represent means ± SEM of 5–7 mice per group and are representative of three independent experiments (*p<0.05; **p<0.01; ***p<0.001).

By week 2 of infection, 1MT treated A/J and B10.A mice showed a higher number of myeloid, lymphoid and plasmacytoid dendritic cells (CD11c^high^CD11b^+^, CD11c^high^CD8^+^ and CD11c^high^B220^+^, respectively) than control mice (Figure 5A). A similar effect on DCs was seen at week 8 of infection when an augmented influx of all dendritic cell subsets were seen in the lungs of 1MT treated resistant and susceptible mice (Figure 5B).

### IDO blockade increases the early influx of T cells to the lungs of susceptible and resistant mice

**Figure 6:**
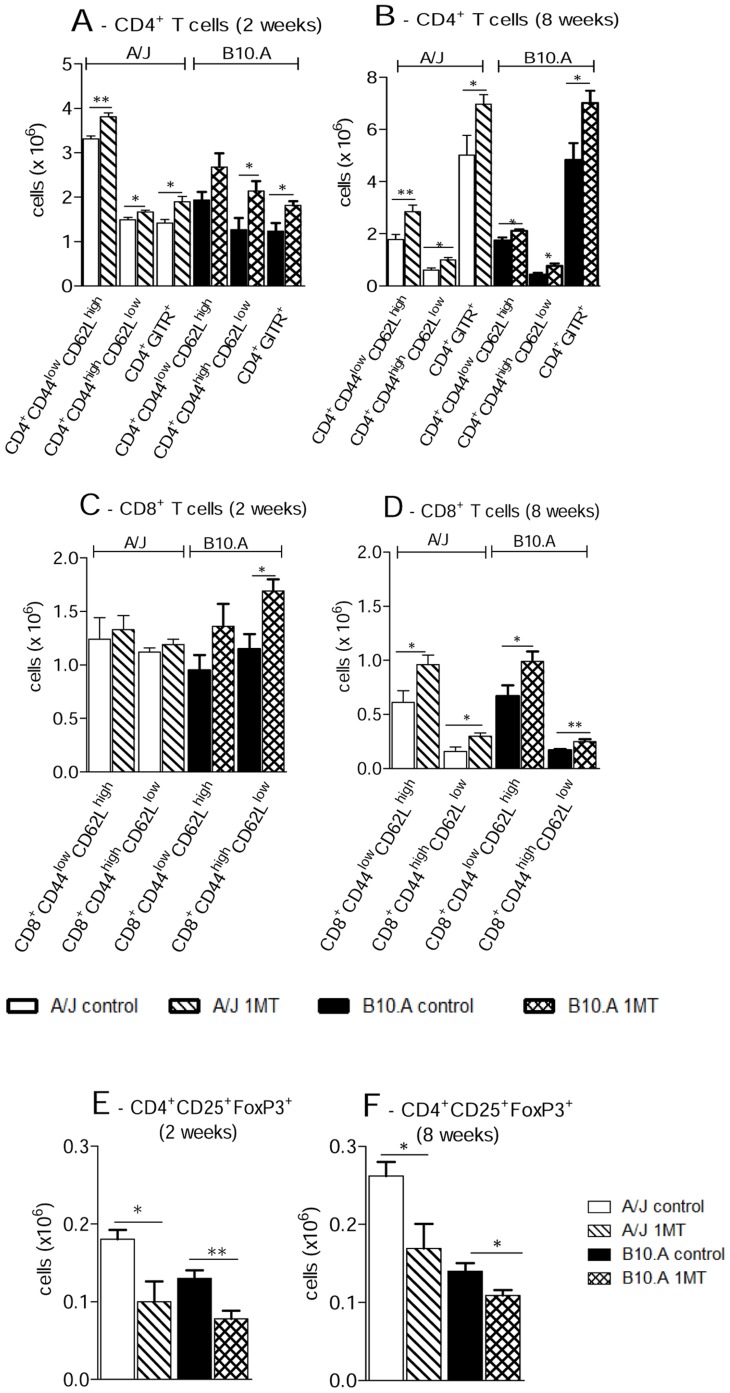
IDO inhibition increases the migration of naïve and effector/memory CD4^+^ and CD8^+^ T cells to the lungs of A/J and B10.A mice, but reduces the number of regulatory T cells. (**A, B, C, D**) Characterization of CD4^+^, CD8^+^ T cells by flow cytometry in the lung infiltrating leucocytes (LIL) from 1MT treated and untreated A/J and B10.A mice inoculated i.t. with 1×10^6^ *P. brasiliensis* yeast cells. At weeks 2 and 8 after infection, lung cell suspensions were obtained and stained as described in Materials and Methods. The acquisition and analysis gates were restricted to lymphocytes. The total numbers of effector (CD44^high^CD62L^low^) and naïve (CD44^low^ CD62L^high^) CD4^+^ and CD8^+^ T cells at weeks 2 and 8 after infection were determined. (**E, F**) To characterize the number of Treg cells in LIL at weeks 2 and 8 weeks of infection, cells positive for surface staining of CD25 and intracellular Foxp3 expression were back-gated on the CD4^+^ T cell population. The data represent the means ± SEM of 6–8 mice per group and are representative of three independent experiments (*p<0.05 and **p<0.01).

Treatment of B10.A and A/J mice with 1MT profoundly affected the influx of inflammatory T cells to their lungs. Increased numbers of CD4^+^ and CD8^+^ naïve (CD4^+^CD44^low^CD62L^high^ and CD8^+^CD44^low^CD62L^high^, respectively) as well as CD4^+^ and CD8^+^ memory/effector (CD4^+^CD44^high^CD62L^low^ and CD8^+^CD44^high^CD62L^low^) T cells were detected at weeks 2 and 8 after infection (Figure 6A, 6B, 6C, 6D). When other activation markers of T cells were studied, the CD4^+^GITR^+^ subpopulation appeared in augmented number in the 1MT treated groups of both mouse strains (Figure 6A, 6B). To verify if 1MT administration affected the presence of Treg cells in the lungs, the number of CD4^+^CD25^+^ T cells expressing Foxp3 was assessed. Indeed, decrease numbers of FoxP3^+^ Treg cells in the lungs of resistant and susceptible mice at both post-infection periods were observed (Figure 6E, 6F).

### IDO inhibition diminishes the production of pulmonary IFN-γ

**Figure 7:**
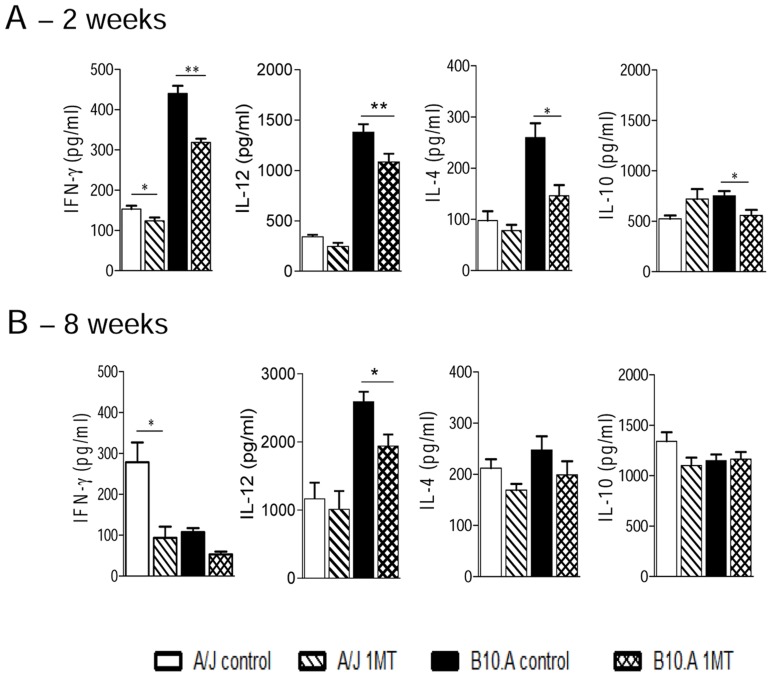
Levels of Th1/Th2 cytokines in the lungs of 1MT treated and untreated A/J and B10.A mice. (**A, B**) At week 2 and 8 after i.t. infection with 1×10^6^ yeast cells of *P. brasiliensis*, lungs from 1MT treated and untreated A/J and B10.A mice were collected, disrupted in 5 ml of PBS and supernatants analyzed for Th1 and Th2 cytokines (IFN-γ, IL-12, IL-4 and IL-10) by capture ELISA. The bars depict mean ± SEM of cytokine levels (6–8 mice per group). The results are representative of three independent experiments (*p<0.05 and ** *p*<0.01).

The presence of cytokines was analyzed in lung homogenates obtained at weeks 2 and 8 after infection. Lower levels of IFN-γ were detected at week 2 of infection in both mouse strains treated with 1MT, but IL-12, IL-4 and IL-10 appeared in lower levels only in B10.A treated mice (Figure 7A). When these Th1/Th2 cytokines were analyzed at week 8, discrete but significant differences were observed after 1MT administration regarding IFN-γ production in A/J, and IL-12 in B10.A mice (Figure 7B). Altogether, the cytokine data demonstrate that 1MT treatment induces an early decrease of the proinflammatory cytokine IFN-γ in both mouse strains and of the inhibitory cytokines IL-4, IL-12 and IL-10 only in susceptible mice.

### IDO inhibition augments production of Th17-associated cytokines and recruitment of PMN leukocytes to the lungs

**Figure 8:**
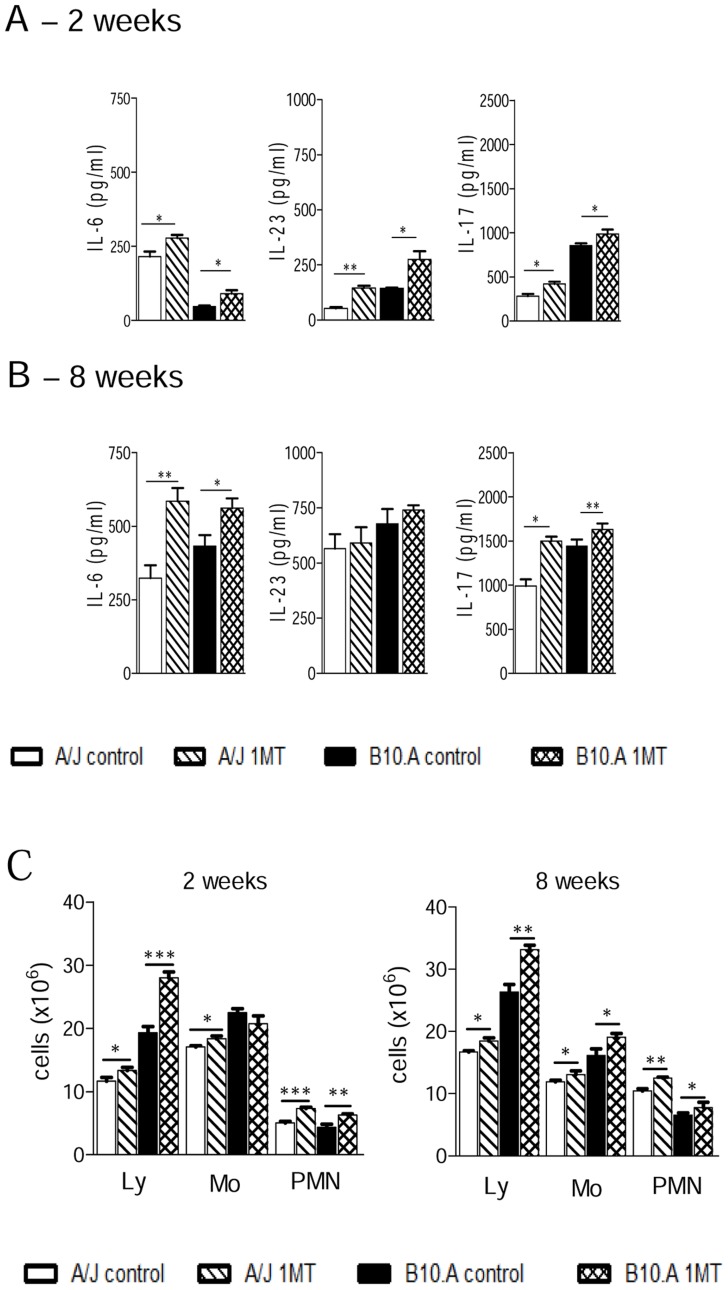
Lungs of 1MT treated A/J and B10.A mice show increased levels of IL-6, IL-23 and IL-17 and concomitant increase of inflammatory cells (lymphocytes, monocytes and neutrophils). (**A, B**) At 2 weeks and 8 after i.t. infection with 1×10^6^ *P. brasiliensis* yeast cells, lungs from A/J and B10.A mice, treated or not with 1MT, were disrupted and supernatants were analyzed for cytokine contents by capture ELISA (IL-6, IL-23 and IL-17). Data are means of cytokine levels ± SEM (6–8 mice per group). The results are representative of three independent experiments. (**C**) Absolute number of leukocyte subsets (macrophages, lymphocytes and PMN neutrophils) in the lung infiltrating leukocytes (LIL) from 1MT treated and untreated A/J and B10.A mice inoculated i.t. with 1×10^6^ *P. brasiliensis* yeast cells. At weeks 2 and 8 post infection, lungs (6–8 mice per group) were excised, washed in PBS, minced, and digested enzymatically. Lung cell suspensions were obtained and cytospin onto glass slides. Cells were stained by the Diff-Quik bloodstain. Data are representative of three experiments with equivalent results (*p<0.05, **p<0.01 and ***p<0.001).

For the assessment of Th17 cell differentiation, we measured cytokines levels in lung homogenates. The Th17-associated cytokines IL-6, IL-23 and IL-17 were synthetized in higher levels in 1MT treated mice at 2 week after infection in both, resistant and susceptible mice (Figure 8A). Compared with early infection, at week 8 an elevated production of cytokines belonging to the Th17 axis was detected in both mouse strains (Figure 8B). However, only the level of IL-6 and IL-17 were higher in 1MT-treated mice than in untreated ones. To better characterize the inflammatory reaction at the site of infection, leukocyte recruitment to the lung tissues of *P. brasiliensis* infected A/J and B10.A mice, treated or not with 1MT, was studied at 2 and 8 weeks postinfection. As can be seen in the Figure 8C, higher numbers of lymphocytes, monocytes and polymorphonuclear neutrophils (PMN) were observed in the lungs of 1MT treated than in their untreated controls in both mouse strains.

### IDO blockade induces increased numbers of innate and adaptive immune lymphocytes expressing IL-17, associated with reduced numbers of cells expressing IFN-γ

**Figure 9:**
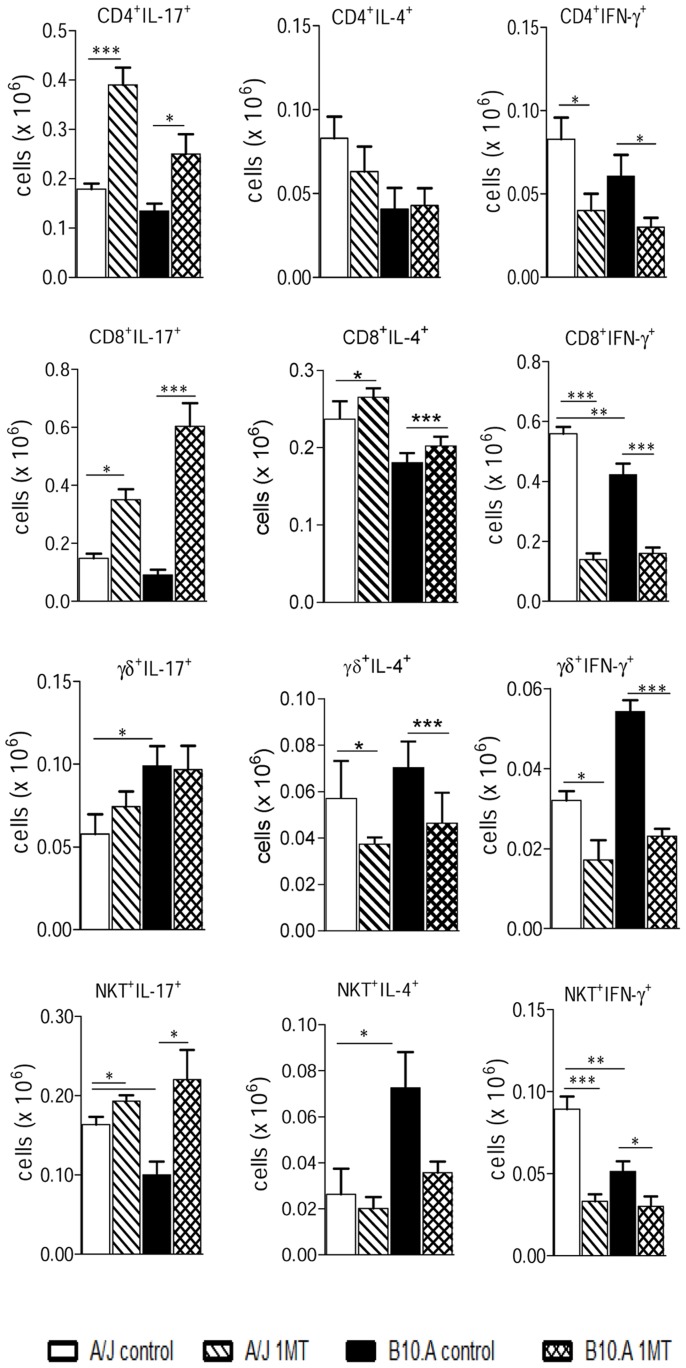
IDO blockade increases the presence of IL-17^+^, but reduces the number of IFN-γ^+^ T cells in the lungs of A/J and B10.A mice. Lymphocytes from lung infiltrating leukocytes (LIL) obtained from groups of 5–6 mice were gated based on forward/side scatters analysis. Gated cells were assessed for CD4^+^, CD8^+^, γδ^+^ and NKT^+^ markers using labeled antibodies. The presence of IL-17^+^, IFN-γ^+^ and IL-4^+^ T cells in the LIL was determined by intracellular cytokine staining at week 2 after infection. Lung cells were stimulated *in vitro* with PMA/ionomycin for 6 h, the last 4 h in the presence of brefeldin A, and subjected to intracellular staining for IL-17, IL-4 and IFN-γ. The results are representative of three experiments with equivalent results (*p<0.05, **p<0.01 and ***p<0.001).

To better clarify the importance of 1MT treatment in the polarization of T cell responses, the phenotypes of IL-17, IFN-γ, and IL-4-producing cells were defined in the inflammatory infiltrates of lungs at week 2 post-infection (Figure 9). These cytokines were assessed by intracellular staining in CD4^+^, CD8^+^, γδ^+^ and NKT^+^ T cells. Compared to control groups, 1MT treated B10.A and A/J mice developed increased numbers of CD4^+^, CD8^+^ and NKT^+^ T cells expressing IL-17 but no differences were observed in γδ^+^ IL-17^+^ T cells. Also, the results with 1MT treated mice showed evident reduction of CD4^+^, CD8^+^, γδ^+^ and NKT^+^ T cells expressing IFN-γ in both mouse strains. No significant differences in CD4^+^, CD8^+^, γδ^+^ and NKT^+^ T cells expressing IL-4 were detected after 1MT treatment (Figure 9). These data demonstrate that 1MT interferes with the T cell phenotypes of resistant and susceptible mice, reducing the Th1 and increasing the Th17 differentiation.

## Discussion

A large number of studies have established that the adequate control of infectious processes and the associated inflammatory reactions require IDO induction and synthesis of tryptophan metabolites. In the present work, we report for the first time the relevant biological role played by IDO in the control of pulmonary paracoccidioidomycosis. The effect of IDO inhibition by 1MT was assessed using in vitro and in vivo assays employing resistant and susceptible mice. We verified that in both mouse strains IDO activity inhibits fungal growth and control T cells expansion, potentiating Th1 and inhibiting Th17 differentiation. The reduced IDO-mediated inflammation was associated with impaired DCs differentiation, reduced T cell responses and concomitant expansion of Treg cells. Interestingly, IDO inhibition did not alter the disease outcome of resistant mice but induced progressive fungal growth and dissemination in susceptible mice resulting in increased tissue pathology and mortality rates. Thus, although IDO activity is not responsible for the divergent susceptibility patterns of A/J and B10.A mice, the early IDO production appears to have a more relevant role in the disease control of susceptible than resistant hosts to *P. brasiliensis* infection.

*P. brasiliensis* infection was more severe in 1MT treated than untreated macrophages of resistant and susceptible mice and this finding was concurrent with decreased production of kynurenines and impaired expression of IDO mRNA. The increased fungal loads could not be attributed to decreased synthesis of NO, one of the most important fungicidal mediators of phagocytes [44], that was detected in elevated levels in 1MT treated cells. In addition, our in vitro studies have also revealed the Th1-inducing property of IDO by its enhancing effects on IL-12 production and its inhibitory activity on Th17 development mediated by the down regulation of IL-6 and TGF-β production [21], [46].

The results of in vivo infection were consistent with the in vitro studies. IDO inhibition was shown to induce increased fungal loads in resistant and susceptible mice concomitantly with increased induction of NO synthesis. This result appears to be contradictory, but several studies have demonstrated that the immunomodulatory roles of IDO and iNOS are cross regulated. The elegant study of Alberati-Giani et al. [47] showed that in IFN-γ activated macrophages NO down modulates the expression of IDO, and the use of iNOS inhibitors increases IDO activity and mRNA expression. In our model, 1MT treatment was shown to reduce IDO activity and expression and this finding was concomitant with significant increase in fungal loads and NO levels. These results lead us to suppose that the increased number of fungal cells through their diverse PAMPs was able to activate PRRs and cell signaling pathways that resulted in increased iNOS expression and NO production that further inhibited IDO expression and activity. However, the increased NO production, was not sufficient to control fungal growth that in untreated macrophages was mediated by both, iNOS and IDO activity. These results have also suggested that the IDO-mediated fungicidal activity is so or even more important than that mediated by NO.

In both mouse strains, 1MT treatment was interrupted at the second week of infection, but only in susceptible mice increased fungal loads were persistently found at later phases (week 8) of infection. This fact indicates a more important effect of early IDO activity in susceptible than in resistant mice. However, we have tested if prolonged IDO inhibition would delay infection severity also in resistant mice. Indeed, 1MT administration during 30 days through subcutaneous pellets led resistant mice to show increased fungal loads at week 8 of infection, indicating that in murine paracoccidioidomycosis IDO exerts a persistent control of fungal growth, but early in the infection IDO activity is more important for susceptible than resistant mice.

The lungs of *P. brasiliensis* infected mice showed increased levels of kynurenines and expression of IDO mRNA that were down regulated by 1MT treatment. Despite the equivalent levels of kynurenines observed in the lungs of infected susceptible and resistant mice, the latter expressed higher levels of IDO mRNA, indicating the existence of distinct regulatory pathways dependent on the diverse immune responses mounted by hosts of different genetic patterns [48], [49]. Indeed, our previous studies have shown that TGF-β dominates the macrophage and DC responses of A/J mice, whereas the IL-12/IFN-γ axis characterizes the responses of B10.A mice [35], [36], [50]. Interestingly, Pallota et al. [48] have elegantly demonstrated a catalytic function for IDO when induced by IFN-γ and a signaling function when TGF-β is the main stimulus, leading us to suppose that IDO could exert diverse activities in resistant and susceptible mice, a hypothesis that we are currently investigating.

Despite the diverse disease outcomes, IDO inhibition induced some phenotypes that were shared by resistant and susceptible mice: the increased migration of DCs and CD8^+^ and CD4^+^ T cells concomitantly with reduced influx of Treg cells to the site of infection. Although not measured, we believe that increased levels of chemokines and expression of chemokine receptors that control DCs migration, were produced by 1MT treated macrophages and mice. Possibly, the inhibited IDO activity induced by 1MT treatment increased the number and activity of immunogenic DCs that promoted increased inflammation and T cell responses. In addition, IDO inhibition led to increased TGF-β and IL-6 synthesis enhancing Th17 differentiation and reducing the expansion of Th1 and Treg cells. It is possible that the decreased IL-12 synthesis was mediated by the anti-inflammatory effect of TGF-β and even IL-6. Indeed, the Th1/Treg-inducing properties of IDO were previously described [19], [20], [21], [22], [48] and here confirmed.

The decreased proliferation of IFN-γ producing cells was always associated with enhanced expansion of IL-17 producing T cells. Interestingly, in both mouse strains IDO blockade increased the synthesis of IL-17 by CD4^+^, CD8^+^ and NKT^+^ lymphocytes and decreased the production of IFN-γ by the same T cell subsets, indicting that IDO exert an effective control on the differentiation of diverse T cell subsets.

Our previous studies in murine paracoccidioidomycosis have shown that the expansion of IL-17 producing cells is controlled by several pathogen recognition receptors (TLR-2, TLR-4, dectin-1) as well as the IL-1R and Toll adaptor protein, MyD88 [51], [52], [53], [54]. Th17 cells can exercise opposing effects in pulmonary paracoccidioidomycosis depending on the experimental settings they are induced. Therefore, the expansion of Th17 cells was shown to be deleterious [51], [52] or protective [45], [53], [54], depending on the concomitant differentiation of other Th cell subsets and the control of Treg cells [45], [51], [52], [53], [54]. In the present study, different disease outcomes were observed despite the equivalent regulatory effects of IDO on the Th1/Th17/Treg immunity developed by both mouse strains. These data lead us to ask why the Th17-exacerbated immunity and the increased neutrophil inflammation have not apparently altered the resistance pattern of A/J mice, but worsened the disease and reduced the survival times of susceptible mice?

The murine model of paracoccidioidomycosis has some particularities that help us to elucidate the results here observed. The resistance and susceptibility patterns of A/J and B10.A mice are manifested late in the course of *P. brasiliensis* infection and the innate phase of immunity is opposed to that expected to protect or damage infected hosts. The innate immunity of resistant mice is mainly governed by a TGF-β-rich anti-inflammatory milieu that leads to early tolerance to fungal growth, elevated expansion of highly suppressive Treg cells and delayed, although efficient, CD4^+^ and CD8^+^ T cell immunity. The rescue of T cell immunity appears to be mediated by the early and concomitant development of myeloid DCs that secret high levels of TNF-α, IL-6 and IL-23 that promote the expansion of Th1 and Th17 cells [36], [45], [51].

The protocol here used to inhibit IDO activity rescued A/J mice from early T cell unresponsiveness and the increased T cell immunity was able to reduce fungal loads to levels observed in untreated mice (week 8 post-infection), without inducing increased tissue pathology. We can suppose that the decreased number of Treg cells here detected increased the efficiency of effector T cells, without affecting their control over excessive inflammation. The marked suppressive activity of Treg cells from A/J mice previously characterized [45] appears to have contributed to this behavior. Their increased production of IL-10 and TGF-β, the increased expression of membrane TGF-β and the enhanced expression of CTLA-4 [45] appeared to have assured the efficient control of inflammation. Despite the non-evident rescue of IDO activity, the elevated levels TNF-α produced by resistant (but not susceptible) mice in response to *P.brasiliensis* infection [34]–[36], [38] appears to have contributed for the sustained anti-inflammatory function of their Treg cells. This interpretation is consistent with previous reports demonstrating that high levels of TNF-α produced by Th17 cells and the elevated expression of TNFR2 by Treg cells are critical for the expansion and phenotype stabilization of Treg cells in an inflammatory environment [55], [56]

We have recently demonstrated that the precocious depletion of Treg cells by anti-CD25 antibodies resulted in decreased fungal burdens and precocious development of T (Th1/Th2/Th17) cell immunity by A/J mice, which, however, maintained their usual pattern of resistance. No sterile immunity or decreased mortality were observed [45]. Here, the precocious T cell immunity and the decreased numbers of Treg cells induced by IDO inhibition appear to have compensated the initial uncontrolled fungal growth resulting in unmodified disease outcome. Thus, once more, we could demonstrate that the immunity developed by A/J mice is robust, hardly modified and only temporarily modulated. Independent of the treatment used [45], [57] at the chronic phase the animals return to their basal behavior.

The early immunity of susceptible mice is mainly dependent on the IL-12/IFN-γ axis of innate immunity that initially controls fungal growth but also induces excessive production of NO and other suppressive mediators that irreversibly suppress CD4^+^ T cell immunity [29], [31], [32], [33], [35], [36], [40], [45]. In contrast to resistant mice, the disease of susceptible mice is easily modulated: administration of rIL-12 induces protective IFN-γ production but also a severe lung inflammation [58]. Pre-immunization leads to sterile immunity, which rescues B10.A mice from precocious mortality [57]. Interestingly, depletion of Treg cells was not able to recover T cell immunity of B10.A mice, but resulted in early and robust inhibition of fungal growth and dissemination that led to regressive disease, diminished tissue pathology (in the lungs and liver) and decreased mortality rates [45]. In the present study, IDO inhibition led to increased fungal burdens that were not controlled despite the increased Th17 cell responses and decreased Treg cells expansion, resulting in disseminated disease and increased mortality rates. These findings demonstrate that B10.A mice can escape from lethal disease mainly based on their robust innate immunity and efficient fungal clearance resulting in regressive disease independently of adaptive immunity. Our data suggest that pathogen-induced inflammation is more deleterious for susceptible mice than immunosuppression or enhanced T cell immunity unable to control fungal burdens.

In agreement with previous findings in experimental candidiasis and aspergillosis [19], [59], [60], the opposed Th1/Th17 expansion and defective Treg differentiation was deleterious to susceptible mice to paracoccidioidomycosis. Our findings demonstrated that their enhanced T cell immunity was not sufficient to overcome the IDO-mediated control of fungal growth in the lungs, avoid fungal dissemination to liver and restrain inflammatory reactions resulting in enhanced mortality. Once more, in murine paracoccidioidomycosis we could verify that disease severity is much more associated with liver than lung pathology [40], [45]. Moreover, in B10.A mice the compromised fungal clearance associated with increased Th17 immunity inadequately controlled by Treg cells resulted in the worst scenario for a host after an infectious process: excessive pathogen growth associated with uncontrolled inflammatory reactions. This appears to explain the increased mortality of B10.A mice. Thus, although host immunity is crucial in eradicating infection, uncontrolled immunological reactivity may contribute toward worsening disease [61], [62], [63]. Importantly, even in some cases of human paracoccidioidomycosis, the severity of disease has been associated with increased or uncontrolled inflammatory responses [64], [65].

In summary, our studies demonstrate for the first time that IDO has an important role in the control of pulmonary paracoccidiodomycosis. Independent of the host genetic pattern, IDO was shown to promote fungal clearance and inhibit immunity and inflammation. Studying the biological functions of IDO enzymes during intracellular infections, Divanovic et al. [16] have confirmed the opposing effects of IDO function. 1MT treatment enhanced the parasite burden of *Toxoplasma gondii* infected mice but did not affect the immune response; in *Leishmania major* infected hosts IDO inhibition decreased parasite loads mediated by increased effector immunity, whereas in herpes simplex virus infection no role for IDO was detected. They concluded that in infectious processes the role played by IDO is pathogen specific. We agree with this conclusion and add a new complexity to these observations. Despite the specific effect of IDO in response to a certain pathogen, the genetic pattern of the hosts and their typical immune reactivity, have also important influence on IDO activity and its role in disease control. As here demonstrated, hosts with different genetic backgrounds and divergent immune responses impose distinct degrees of importance to IDO activity and its influence in the control of infection.
